# Interrogating the Impassable: A Case Series and Literature Review of Unilateral SPECT-CT Groin Visualization in Men With Penile Cancer

**DOI:** 10.3389/fsurg.2022.882011

**Published:** 2022-05-18

**Authors:** Jonathan S. O'Brien, Jiasian Teh, Brian D. Kelly, Kenneth Chen, Todd Manning, Marc Furrer, Justin Chee, Nathan Lawrentschuk

**Affiliations:** ^1^Division of Cancer Surgery, Peter MacCallum Cancer Center, Melbourne, VIC, Australia; ^2^Sir Peter MacCallum Department of Oncology, The University of Melbourne, Melbourne, VIC, Australia; ^3^Department of Urology, The Royal Melbourne Hospital, Melbourne, VIC, Australia; ^4^Young Urology Researchers Organization (YURO), Melbourne, VIC, Australia; ^5^Department of Urology, Singapore General Hospital, Singapore, Singapore; ^6^Department of Surgery, Austin Health, The University of Melbourne, Melbourne, VIC, Australia; ^7^MURAC Health, East Melbourne, VIC, Australia; ^8^EJ Whitten Prostate Cancer Research Center at Epworth Healthcare, Melbourne, VIC, Australia

**Keywords:** penile cancer, SPECT-CT, sentinel node (SN), dynamic sentinel lymph node biopsy (DSLNB), minimally invasive surgeries (MIS)

## Abstract

Penile squamous cell carcinoma (SCC) is a rare malignancy, which is known to invade local inguinal lymph nodes prior to progressing to the pelvis. Dynamic sentinel lymph node biopsy (DSLNB) is a standard for the minimally invasive assessment of lymphadenopathy in patients with subclinical groin metastasis. Hybrid ^99m^Tc Single-Photon Emission Computed Tomography (SPECT-CT) has been shown to increase the accuracy of identifying first draining “sentinel” nodes (SN). Unilateral inguinal visualization on SPECT-CT is a rare presentation, which may increase the likelihood of a false negative SN biopsy. Retrospective analysis from three-penile cancer uro-oncologists in Melbourne, Australia identified 78 groins undergoing DSLNB for intermediate/high risk primary disease. Unilateral SPECT-CT results were observed in four patients suggesting a functional pattern of lymph diversion. Analysis confirmed malignancy (*n* = 2), sarcoidosis (*n* = 1), and evidence of local inflammation in SPECT-CT negative groins. Findings re-iterate the role of SPECT-CT a pre-operative adjunct. Experienced multimodal groin assessment using palpation, SPECT-CT, lymphoscintigraphy, and blue dye tracking remains paramount. Unilateral SN on pre-operative SPECT-CT in men with intermediate/high-risk penile SCC should elicit a higher degree of clinical suspicion. We recommend a low threshold for recommending radical inguinal lymph node dissection (ILND) for groins refractory to minimally invasive assessment.

## Introduction

Penile squamous cell carcinoma (SCC) accounts for <1% of male malignancies in developed nations (1). The presence of lymph node metastasis is a paramount prognostic indicator for men with high-risk primary disease (2). In the past, durable disease free survival required penile amputation as well as bilateral radical inguinal lymph node dissection (ILND). However, in men with non-metastatic disease, radical ILND results in long-term morbidity with limited oncological benefit (3).

Dynamic sentinel lymph node biopsy (DLSNB) is the standard of care for the minimally invasive assessment of patients with sub-clinical groin metastasis (4). Preoperative Single-Photon Emission Computed Tomography (SPECT-CT) has been shown to increase the accuracy of identifying the first first draining sentinel nodes (SNs) by correlating the patient's lymphatic anatomy with plain CT imaging (5).

The pattern of unilateral inguinal lymph node visualization on SPECT-CT is a rare occurrence that presents a difficult clinical challenge. Theories suggest that lymphadenopathy within a SN can occlude lymphatic flow causing fluid to be redirected to a contralateral “neo-sentinel node” via redundant vessels at the base of the penis (6). Reliance on unilateral SPECT-CT findings may increase the likelihood of a false-negative DSLNB (7). Here, we present a case series highlighting practical management strategies and histopathological patterns of unilateral SPECT-CT visualization for men with intermediate/high-risk penile SCC.

## Materials and Methods

**Figure 1:**
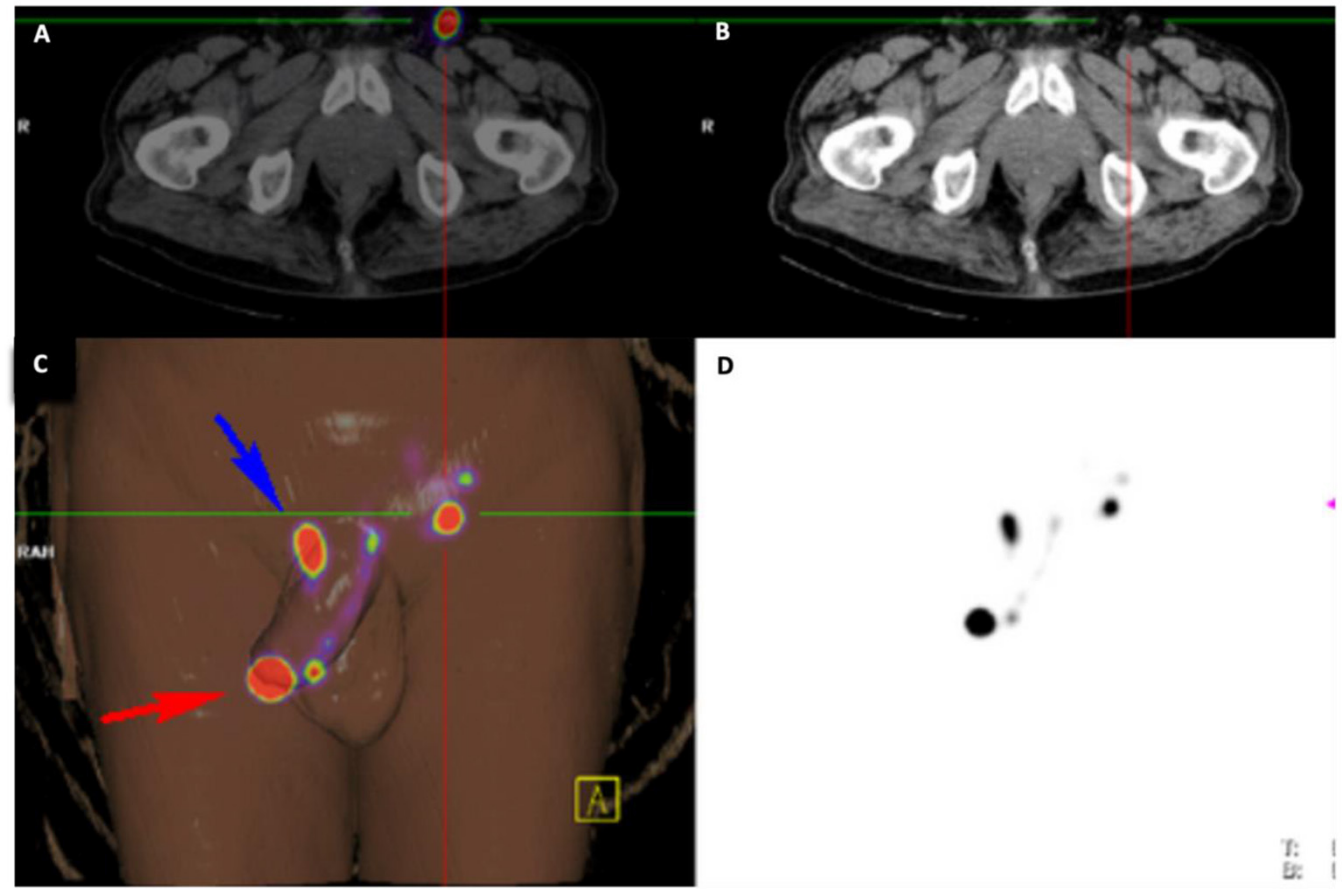
Imaging Overlay for Patient B. The primary penile lesion and left inguinal node is identified with radiotracer hold up at the base of the penis using **(A)** ^18^FDG PET-CT, **(B)** conventional CT, **(C)** 3-dimensional digital reconstruction amalgamating information from all imaging modalities, and **(D)** ^99m^Tc-lymphoscintogram.

Multi-institution ethical approval was obtained, and retrospective data was collected from three uro-oncologists with sub-specialist experience in penile cancer. Patients with (≥ pT1G2) intermediate/high-risk (pT1G2) penile SCC underwent standard staging with physical examination, inguinal ultrasound (USS) with attempt of fine-needle aspiration (FNA) as recommended by European Association of Urology (EAU) guidelines (2, 4). Further staging was performed with either conventional pelvic CT or ^18^FDG PET-CT as per hospital availability and surgeon preference. A total of 28 patients with cN0 groins underwent pre-operative lymphoscintigraphy with intradermal injection of 50–60 MBq ^99m^Tc nanocolloid proximal to the tumor 4–18 h prior to DSLNB. Planar lymphoscintigraphy was carried out and static images acquired at 30 min and 120 min [8]. Low dose CT (40 mAs, 130 Kv) and SPECT (256 × 256 matrix, two heads, 180 degree rotation with 20 views/head and 30 s/view) were performed 90 min post injection using a dual-head SPECT/CT gamma camera (Symbia T6, Siemens A.G., Darmstadt Germany). Attenuation and scatter correction was carried out and fused axial SPECT-CT images rendered using orthogonal multiplanar reconstruction using MIM software package (version 6.1, MIM Software Inc, Cleveland OH, USA). Images were formally reviewed by a nuclear medicine specialist and interventional radiologist for tracer uptake and anatomical location (Figure 1). A radioactive region was defined as an SN, if afferent lymphatic channels were visible, if the inguinal radioactivity was solitary, or it was the most proximal in a lymph node chain. The location of SN was confirmed using a portable gamma camera and marked on the skin with a pen (8).

Four cases of unilateral groin uptake were identified on SPECT-CT. Pre-operatively these patients were counselled on SPECT-CT findings and were offered to delay surgery for repeat lymphoscintigraphy. A shared decision was made to proceed for surgery with the understanding that superficial modified lymphadnectomy (SML) may be performed in the non-visualized groin. Patients were re-examined intraoperatively and standard DLSNB was performed. At the beginning of the case, 1 ml of methylene blue dye was injected intradermally into the proximal tumor base. SNs were identified using a combination of skin markings, intra-operative gamma probe, dye visualization (9). SNs were bisected, fixed with formalin, embedded in paraffin, further sectioned, and stained with hematoxylin and eosin. Formal review of all tissue specimens was performed by a high-volume genitourinary pathologist. Patterns of staging, operative techniques, and pathology were assessed and summarized in Table 1.

**Table 1:** Patient Outline.

	**Age**	**Penile tumor pathology**	**Pre-operative staging**								**Intraoperative**				**Lymph node malignancy**			
			**USS**	**Pelvic CT**	**^**18**^FDG PET-CT**	**SPECT CT**	**Gamma probe**	**Methylene blue**	**DSLNB**	**ILND**
			**R**	**L**	**R**	**L**	**R**	**L**	**R**	**L**	**R**	**L**	**R**	**L**	**R**	**L**	**R**	**L**
A	65–70	SCC pT2	–	–	–	–			+	–	+	+	+	+	1/1	1/1	6/10	4/12
B	70–75	SCC pT1b	–	–	–	–	–	–	–	+	–	+	–	+		1/2	2/13	7/20
C	75–80	SCC pT1b	–	–	–	–			+	–	–	+	–	+		0/1	–	–
D	55–60	SCC pT2	+	+	+	+			+	–	+	+	+	+	0/1	0/3		

A literature review was performed according to Preferred Reporting Items for Systematic Review and Meta-Analyses (PRISMA) guidelines (10). Web of Science (with MEDLINE), Embase, and Cochrane databases were searched for English language articles in peer reviewed publications published between 1980 and 2022. The search terms used were “penile cancer”, “lymph node biopsy”, “sentinel node”, “minimally invasive” alone and in combination. A total of 1,060 studies were identified from an initial search, of which 315 were excluded for duplicated reporting. All 745 studies were screened and 638 were excluded by screening titles and abstracts. The remaining 107 original studies were reviewed by carefully screening the full texts, after which 104 were eliminated due to lack of eligible data. Three studies were included in the review.

## Patient A

A man in his late 60s was referred with an 18-month history of a painless non-healing ulcer limited to his glans. Examination demonstrated a 3 cm ulcerated lesion with contact bleeding highly suspicious for SCC and no palpable lymphadenopathy. Staging with inguinal USS ultrasound and pelvic CT was negative for evidence of metastatic disease and pre-operative SPECT-CT identified a unilateral inguinal node on the right. Partial penectomy was performed and intraoperative gamma probe assessment identified bilateral SNs, which were stained with methylene blue. Histopathology of the primary lesion demonstrated pT2 disease and bilateral SNs were positive for SCC. The patient proceeded for bilateral open-radical ILND. Formal pathology confirmed metastatic disease in bilateral nodes with extra-capsular extension. Post-operative re-staging FDG-PET demonstrated high volume pelvic lymphadenopathy that was not present on initial conventional CT. His medical comorbidities limited further surgery and palliative chemotherapy was commenced.

## Patient B

A man in his mid 70s was referred by his local urologist for voiding difficulty and suspicious phimosis. A palpable mass was found on his glans, and examination of his inguinal region was clinically normal. Staging pelvic CT and ^18^FDG PET-CT demonstrated cN0 disease prior to referral to our service. Pre-operative inguinal USS and SPECT-CT identified a unilateral left inguinal SN with significant tracer hold up at the right base of penis (Figure 1). He proceeded to have a glansectomy and left groin DSLNB. Gamma probe and intraoperative blue dye mapping identified a SN for minimally invasive excision. Histopathology from the tumor was confirmed as pT1b with metastatic SCC identified in the with no ECE. The patient was counseled on the presence of left groin metastasis and that DSLNB could not be performed on his right groin. A shared decision was made to proceed for bilateral radical ILND which confirmed bilateral inguinal lymph node metastases with extra nodal extension. Multidisciplinary review of his case recommended to proceeding for adjuvant chemotherapy. He was referred for paclitaxel, ifosfamide, cisplatin (TIP), and died from pneumonia shortly after completing his third treatment cycle.

## Patient C

A man in his late 70s was referred by a dermatologist for relapsed penile SCC 10 years post excisional biopsy and topical therapy with 5-flurouracil (11). Physical examination demonstrated a grossly inflamed 2 cm glans ulcer and non-palpable inguinal nodes. Staging ^18^FDG PET-CT demonstrated no evidence of metastatic disease. Staging USS and pelvic CT demonstrated no evidence of metastatic disease. Pre-operative SPECT-CT demonstrated a unilateral right SN. He proceeded for excisional biopsy, glans resurfacing, and split skin graft for the primary tumor and left DSLNB. Intraoperative re-palpation and gamma probe assessment of his right groin were negative. Given the significant inflammation of the tumor, an intra-operative decision was made to await pathology with the aim to recommending proceeding to radical ILND. Histopathological analysis demonstrated pT1b lesion with clear margins and inflammation only in the left groin. Multidisciplinary recommendation was to offer right radical ILND. After appropriate counseling and consideration of multiple medical comorbidities, a shared decision was made for right groin surveillance. The patient remained disease free for 5 years and was discharged from our service.

## Patient D

A man in his mid 50s with a history of sarcoidosis was referred by his primary care physician with pT1 SCC post punch biopsy. Pre-operative plain CT demonstrated extensive bilateral inguinofemoral lymphadenopathy with a 13 mm left inguinal node that was not palpable due to body habitus. Pre-operative FNA demonstrated inconclusive pathology. Staging pelvic CT identified extensive bilateral lymphadenopathy. Pre-operative SPECT-CT identified a unilateral right SN. He proceeded for concurrent partial penectomy and excision of the right node using standard DSLNB. Intraoperative positioning allowed for palpation of a 1.5 cm left inguinal node. Gamma probe assessment was equivocal over the palpable lesion. SML and blue dye tracking was used to excise the node. Pathology demonstrated a pT2 primary lesion with bilateral inguinal sarcoidosis. The patient was prescribed systemic corticosteroids for a relapse of sarcoidosis. He is currently under surveillance with interval CT and clinical review. He remains disease free after 4 years.

## Discussion

Early identification of inguinal disease combined with ILND remains critical for survival in men with intermediate to high-risk penile cancer (2). Lymphatic metastasis occurs in a predictable pattern, yet accurate assessment of cN0 groins remains a challenge. Physical examination is often limited by patient body habitus and conventional imaging modalities including pelvic CT/MRI and ^18^FDG PET-CT cannot reliably assess disease <10 mm (12).

EAU and NCCN guidelines recommend DSLNB with pre-operative SPECT-CT for assessing inguinal metastases in men with impalpable disease (2, 13). However, concerns over false negative and a difficult learning curve has limited adoption to specialist centers (14).

**Figure 2:**
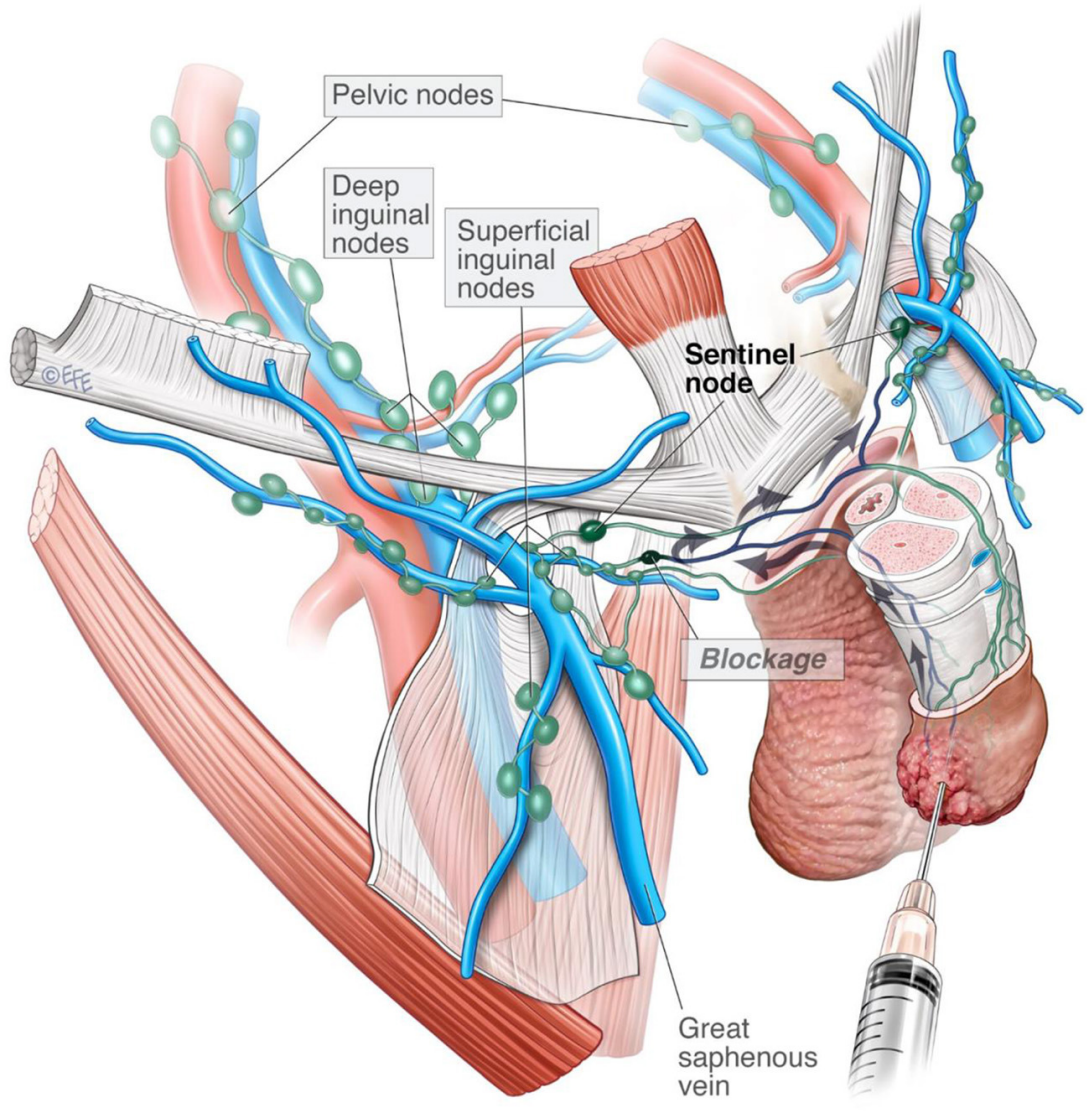
Artistic rendering of re-routed lymphatic drainage.

This case series reports on a rare observation in the context of an uncommon malignancy. Unilateral inguinal visualization on initial lymphoscintigraphy was observed in 5% (*n* = 4) of assessed groins. Half of SPECT-CT silent groins (*n* = 2) were found to contain occult metastases. This observation lends support to previous theories of pathological re-routing of lymphatic drainage to a “neo-SN” in the contralateral groin (6) (Figure 2). Unilateral lymphatic routing in patients C and D were suggestive of local and systemic inflammatory processes, respectively. The literature considers other causes for this observation including previous inguinal surgery, inconsistencies in radiotracer administration, inflammation, lymphoma and physiological unilateral drainage (8, 15).

Previous groups have observed unilateral groin visualization in 12–44% of patients undergoing preoperative SPCT-CT (Table 2). In these studies, 52% (*n* = 34/65) of patients were managed with surveillance. Pathological outcomes are reported in a total of 30 groins. Repeat DSLNB or superficial groin exploration was reported in 83% (*n* = 25) of cases. Radical inguinal dissection was reported by only one group and represents 17% (*n* = 5) of the pathology cohort. Overall, 10% (*n* = 3) groins sampled were positive for malignancy. Long-term follow-up identified recurrence in 1/34 in men undergoing surveillance and 1/30 in the pathology group.

**Table 2:** Summary of SN non-visualization.

**Author**	**Cases** **(*N*)**	**SN** **(*N*)**	**Unilateral Non-visualization** **(*N*, %)**	**Management**	**Malignant pathology** **(Total groins)**	**Recurrence** **(Total groins)**	**Average** **Follow up (months)**
Current study	41	78	4 (5)	DSLNB *n* = 2 ILND *n* = 1 SML *n* = 1 Surveillance *n*=1	1/2 1/1 –	0/2 0/1 0/1	42
Sahdev et al. (8)	166	332	20 (12)	Re-attempt DSLNB *n*=6 SML *n*=8 Surveillance *n* = 6	0/6 0/8 –	0/6 1/8 0/6	36 41.5
Kirrander et al. (14)	55	111	22 (44)	Exploration *n* = 8 ILND *n* = 5 Surveillance *n* = 9	1/3 1/5 –	N/A	21 (median)
Kroon et al. (15)	123	246	23^*^ (19)	Exploration *n* = 8 Surveillance *n* = 19	1/8 –	0/8 1	52 (median)

* *2 patients had bilateral non-visualization (n = 4) and data was pooled in further analysis*.

Our experience managing men with unilateral groin visualization offers several learning points. Patient A demonstrates that the standard bilateral DSLNB assessment can be successfully carried out. It is possible that delayed tracer accumulation or intraoperative positioning helped restore bilateral lymph drainage on the table. Delayed re-establishment SN visibility was reported by Sahdev et al. who found that re-attempt at SPECT-CT was successful for 87% (*n* = 6/7) patients (8).

Patient D demonstrates that intraoperative re-examination and blue dye tracking may be a useful adjunct for groins that fail to adequately uptake radiotracer. Multimodal assessment with intraoperative re-palpation, radiotracer detection, and lymphatic mapping was undertaken by Kirrander and colleagues for 8/22 groins and 3 SNs were identified (15). These results are further supported by group lead by Kroon who explored 8/23 groins using afferent blue dye mapping and were able to identify 4 SN (14). Penile cancer is known to progress rapidly, short delays from pre-operative staging to surgical intervention may allow a previously subclinical lesion to become palpable.

There remains an ongoing conversation within the penile SCC community on the optimal assessment of lymph nodes in the setting of cN0 disease. ^18^FDG PET-CT is limited by its spatial resolution and has limited sensitivity for metastases <10 mm. In addition, FDG avidity from physiologically reactive lymph nodes from an infected primary lesion can mimic malignancy. In a meta-analysis of seven studies by Sadeghi et al. found pooled ^18^FDG PET-CT sensitivity was only 56.5% (95% *CI*: 34.5%−76.8%) in the context of cN0 disease (16). Furthermore, updated EAU and NCCN guidelines do not recommend upfront ^18^FDG PET-CT for staging cN0 disease (2, 13).

Interpretation of this study is limited by retrospective data, small numbers, and inherent selection bias. However, this patient series suggests that unilateral SN non-visualization presents a concern for occult metastasis. Findings re-iterate the role of SPECT-CT as a pre-Operative adjunct. It cannot replace experienced multimodal groin assessment of palpation, SPECT-CT, lymphoscintigraphy, and blue dye tracking. Patients with intermediate to high-risk penile SCC and a groin that fails to uptake radiotracer should be managed with a heightened degree of suspicion. We have adopted a lower threshold for recommending radical ILND for men on a curative pathway with non-visualized unilateral groin.

## Data Availability Statement

The raw data supporting the conclusions of this article will be made available by the authors, without undue reservation.

## Ethics Statement

The studies involving human participants were reviewed and approved by Peter MacCallum Cancer Centre. Written informed consent for participation was not required for this study in accordance with the national legislation and the institutional requirements.

## Author Contributions

JO'B: methodology, investigation, data analysis, and writing—original draft. JT: investigation and analysis. BK, KC, TM, and MF: review and editing. JC and NL: conceptualisation, study design, and supervision. All authors contributed to the article and approved the submitted version.

## Conflict of Interest

The authors declare that the research was conducted in the absence of any commercial or financial relationships that could be construed as a potential conflict of interest.

## Publisher's Note

All claims expressed in this article are solely those of the authors and do not necessarily represent those of their affiliated organizations, or those of the publisher, the editors and the reviewers. Any product that may be evaluated in this article, or claim that may be made by its manufacturer, is not guaranteed or endorsed by the publisher.
